# The genetic basis of divergent melanic pigmentation in benthic and limnetic threespine stickleback

**DOI:** 10.1038/s41437-024-00706-0

**Published:** 2024-07-24

**Authors:** Elizabeth Tapanes, Diana J. Rennison

**Affiliations:** School of Biological Sciences, Section of Ecology, Behavior and Evolution, University of California San Diego, La Jolla, CA USA

**Keywords:** Genetics, Evolution

## Abstract

Pigmentation is an excellent trait to examine patterns of evolutionary change because it is often under natural selection. Benthic and limnetic threespine stickleback (*Gasterosteus aculeatus*) exhibit distinct pigmentation phenotypes, likely an adaptation to occupation of divergent niches. The genetic architecture of pigmentation in vertebrates appears to be complex. Prior QTL mapping of threespine stickleback pigmentation phenotypes has identified several candidate loci. However—relative to other morphological phenotypes (e.g., spines or lateral plates)—the genetic architecture of threespine stickleback pigmentation remains understudied. Here, we performed QTL mapping for two melanic pigmentation traits (melanophore density and lateral barring) using benthic-limnetic F_2_ crosses. The two traits mapped to different chromosomes, suggesting a distinct genetic basis. The resulting QTLs were additive, but explained a relatively small fraction of the total variance (~6%). QTLs maps differed by F_1_ family, suggesting variation in genetic architecture or ability to detect loci of small effect. Functional analysis identified enriched pathways for candidate loci. Several of the resulting candidate loci for pigmentation, including three loci in enriched pathways (*bco1*, *sulf1*, and *tyms*) have been previously indicated to affect pigmentation in other vertebrates. These findings add to a growing body of evidence suggesting pigmentation is often polygenic.

## Introduction

Across the animal kingdom, pigmentation serves as an important communication signal (or disruptor) intra- and interspecifically and thus is often under natural and sexual selection (Cuthill et al. 2017; Hubbard et al. 2010; Orteu and Jiggins 2020; Protas and Patel 2008). Pigmentation and patterning are critical for many key biological functions or interactions–including mate choice, thermoregulation, microbial resistance, crypsis, toxicity warning, and mimicry (Cuthill et al. 2017; Protas and Patel 2008). These signals are often generated through a combination of pigment cells and reflective structures, and both pigmentation and patterning are under strong genetic control (Jablonski and Chaplin 2017; Lopes et al. 2016; Luo et al. 2021; Nachman et al. 2003). As such, animal pigmentation genetics has emerged as a robust model system to study the phenotype-genotype relationship. The interest in this trait has led to the identification of hundreds of loci and pathways involved in vertebrate pigmentation (Elkin et al. 2022; Hidalgo et al. 2022; Kelsh 2004; Lynn Lamoreux et al. 2010). However, studies on the genetic basis of pigmentation are often biased toward certain taxonomic groups (e.g., mice, humans), populations, and/or candidate loci (e.g., *mc1r*, *agouti*) (Elkin et al. 2022; Tapanes et al. 2022)). For example, ~70% of known pigmentation candidate genes emerged from mammalian studies, but only 5% are associated with fish (Elkin et al. 2022). Additional taxa and loci must be studied and identified to gain a more comprehensive and unbiased understanding of the genetic architecture of pigmentation.

Through characterization of the genetic architecture of pigmentation, we gain insight into evolutionary phenomena, including the predictability of phenotypic and genotypic evolution as well as the origins of adaptive genetic variation (Cuthill et al. 2017; Elkin et al. 2022; Martin and Orgogozo 2013). So far, work suggests that often the same loci are co-opted across vast evolutionary scales to produce convergent pigmentation phenotypes (Crawford et al. 2017; Lamason et al. 2005; Miller et al. 2007; Saenko et al. 2015)). For example, *oca2* underlies melanin deficiency in humans and corn snakes. Frequent identification of major effect genes (e.g. *mc1r*) in studies of specific populations (i.e., mice, European people) led to the assumption that pigmentation has a simple genetic architecture, with mutations of large effect generating key phenotypes (Hubbard et al. 2010; Protas and Patel 2008; Quillen et al. 2019). However, recent evidence suggests the underlying genetic architecture of this trait may frequently be complex (highly polygenic) (Anderson et al. 2009; Jones et al. 2018). Further, pigmentation can exhibit rapid phenotypic and genetic change, quickly evolving within a handful of generations, and adapting to new environments (Barrett et al. 2019; Jones et al. 2018). To fully understand pigmentation in light of evolutionary change, we must be able to robustly characterize the genetic architecture in more than a handful of organisms.

The threespine stickleback (*Gasterosteus aculeautus*; hereafter referred to as ‘stickleback’) offers an opportunity to study key evolutionary processes and patterns, such as—evolutionary predictability and adaptation, and with ample genetic resources it is possible to characterize genetic architecture of adaptive traits. Marine stickleback repeatedly and rapidly colonized newly formed freshwater habitats at the end of the Pleistocene (≈12,000 years ago) (Bell and Foster, 1994). Within lakes and streams, stickleback independently adapted to the local ecological conditions–often diverging along a benthic-limnetic axis. Within a handful of lakes there has been the evolution of sympatric benthic and limnetic ecotypes which utilize the littoral and pelagic regions of the lakes, respectively (Schluter and McPhail 1992). These sympatric ecotypes have diverged both genetically and phenotypically in response to their divergent niches (Jones et al. 2012; Peichel et al. 2001; Schluter and McPhail 1992). Notably, phenotypic divergence involves suites of trophic (Schluter and McPhail 1992) and defensive traits (Vamosi and Schluter 2004). However, the ecotypes have also diverged in several pigmentation traits (Clarke and Schluter 2011; Greenwood et al. 2011; Gygax et al. 2018; Miller et al. 2007).

Limnetic fish exhibit greater ventral pigmentation (Miller et al. 2007) and more lateral barring than benthic fish (Greenwood et al. 2011). Increased brightness has been associated with increased use of limnetic resources (French et al. 2018; Bolnick and Ballare 2020; Lavin and McPhail, 1986). Additionally, green pigmentation in the dorsal region is more prevalent in benthic fish (Clarke and Schluter 2011; Gygax et al. 2018). Males of each ecotype also differ in their nuptial coloration—limnetic males exhibit redder throat patches relative to benthic stickleback, and often have an intensely blue iris (Boughman 2001). Differences in pigmentation are predicted to be adaptive as there is covariance with the spectral qualities of each ecotype’s primary habitat (littoral vs. pelagic) (Clarke and Schluter 2011; Rennison et al. 2016); and the preferred nest sites of the two ecotypes also differ in spectral quality (Boughman 2001). The visual sensitivities of the two ecotypes also exhibit divergence (Boughman 2001; Rennison et al. 2016), suggesting differential perception of intra- and inter-specific pigment signals could contribute to pigmentation divergence (Boughman 2001). Further, there are distinct predation regimes between the habitats (Vamosi and Schluter 2004) and differential exposure to a vertebrate predator has been found to be associated to divergence of pigmentation (Gygax et al. 2018), suggesting selection due to crypsis may also drive the evolution of pigment differences.

Quantitative trait mapping (QTL) studies for some stickleback pigmentation traits have successfully identified candidate genes or genomic regions. So far, work using marine-freshwater pairs has characterized candidate regions for two pigmentation traits: lateral barring and ventral melanism (Greenwood et al. 2011; Greenwood et al. 2012). Candidate regions have also been found for both male and female nuptial coloration, specifically male red throat chroma was mapped in a benthic-limnetic pair (Malek et al. 2012) and red throat and pelvic spine pigmentation in females from allopatric stickleback populations (Yong et al. 2015). Yet, in general, we know little about the genetic architecture of pigmentation traits of stickleback or how the genetic architecture varies across populations. More than 1000 QTL have been identified for various stickleback phenotypes (behavioral, morphological, or life history), but only 20 (1.7%) are associated with pigmentation traits. Furthermore, of the 27 threespine stickleback QTL studies included in a 2017 meta-analysis of stickleback QTL, only four studies mapped pigment traits (Peichel and Marques 2017). However, gene expression studies have also been useful in the identification of pigment-associated genes (McKinnon et al. 2022).

Here, we conducted a QTL mapping study of two melanin-based pigmentation phenotypes—melanophore density and lateral barring using threespine stickleback benthic-limnetic F_2_ crosses. We focused on these traits as there is experimental evidence that melanism and lateral barring are adaptive phenotypes, diverging in response to differential predation pressures (Gygax et al. 2018), which aids in vertebrate predator avoidance. Once candidate regions were identified, functional enrichment analyses were used to further characterize the resulting loci.

## Materials and Methods

Four F_1_ crosses were made in the Spring of 2011 using four benthic females and four limnetic males collected from Paxton Lake on Texada Island, British Columbia, Canada. These F_1_ families were reared in 100 L tanks under standard laboratory conditions for nine months. Once the fish reached reproductive maturity in the Spring of 2012, each F_1_ family was split between a pair of semi-natural ponds (*n* = 8 ponds), which were located on the University of British Columbia campus in Vancouver, Canada. The pond-rearing facility and cross-design have been previously described (Arnegard et al. 2014; Rennison et al. 2019). Each pond was 25 m × 15 m, and included a vegetated littoral zone as well as a deep-water habitat of 6 m (S1). The ponds contained typical natural food resources and invertebrate predators. Once in the ponds, the F_1_ fish reproduced naturally from May – July 2012. In the fall of 2012, a sample of the resulting juvenile F_2_ fish was taken from each pond (S2). Immediately following collection by minnow trap or net, fish were euthanized using MS-222 and preserved in 95% ethanol. The fish were fin-clipped for DNA extraction and subsequent genotyping.

### Pigmentation variation

**Fig. 1:**
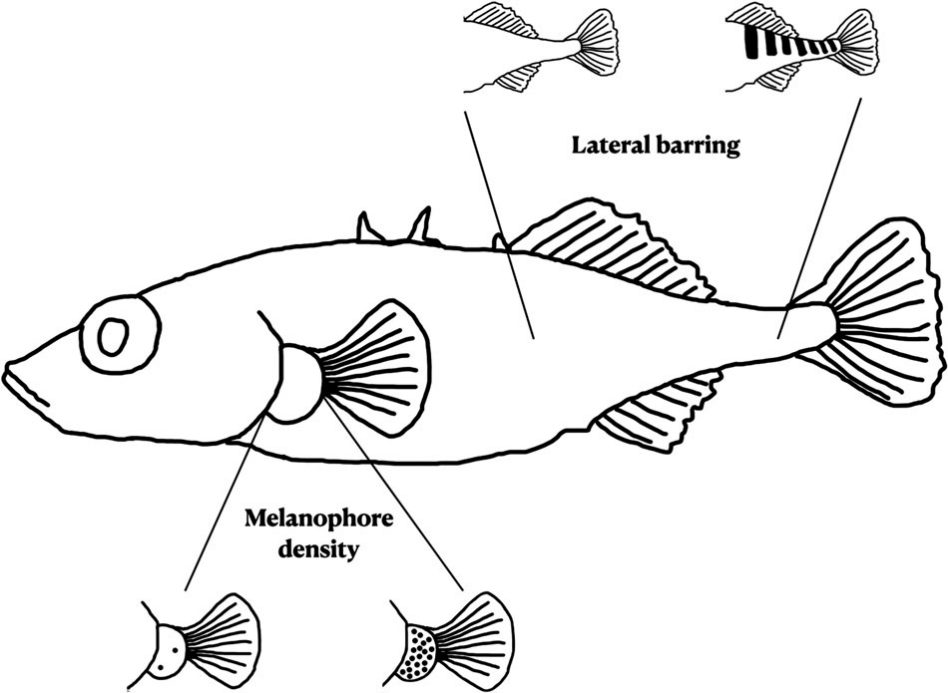
Stickleback phenotypes scored in this study: melanophore density of the fin junction and the degree of lateral barring along the flank.

We phenotyped two pigmentation traits in our F_2_ sample (*N* = 400)—melanophore density and lateral barring (Fig. 1). Using Adobe Photoshop, we estimated melanocyte density by manually counting the number of visible melanocytes on the fin junction using the ‘count tool’ and dividing the resulting count value by the total area (in mm) (*similar to* (Miller et al. 2007)) (S3). We estimated lateral barring by converting each photo to black and white and estimating the absolute difference between a light and dark patch on the ventral flank (S4). This was done by selecting two squares 11 × 11 pixels in size, and placing the first point on the dark bar superior to the start of the anal fin. If the fish exhibited a clear barring pattern, the second square was placed on the brighter region between the two dark bars. If the fish lacked a clear barring pattern, we selected two squares at an average distance typical of two dark bars (≈1.12 mm) and took the absolute difference between both points. To account for potential differences in lighting conditions across each photo, we divided the absolute difference by the average grey value of the photo, and then multiplied that number by 100 (*similar to* (Greenwood et al. 2011)).

Since some previous pigmentation work has relied on scoring phenotypes on living fish (Greenwood et al. 2011; Gygax et al. 2018; Clarke and Schluter 2011), we first sought to test the validity of scoring melanic pigmentation on stained and preserved fish. Our verification sample included live wild marine (Sooke, Courtenay, Sayward estuaries) and freshwater (Mohun, Comox, and Muchalat lakes) populations (*N* = 170) that were collected in British Columbia in Spring 2021. We photographed fish prior to euthanasia against an X-rite color checker passport using a Nikon D500 mounted with the AF-S DX NIKKOR 16–80 mm f/2.8–4E ED VR lens (S5). We set a manual white balance by calibrating the camera with a grey standard in the field. All fish were photographed against a blue background following established protocols (Gygax et al. 2018; Stevens et al. 2007). Instead of melanophore density, we scored brightness as a proxy for overall pigmentation as brightness is a good measure of the relative prevalence of black pigmentation. This was necessary because the melanophore density was difficult to score on many of the marine individuals due to the presence of other colors (e.g., silver iridescence) and bony elements. We estimated an individual’s brightness using the Red-Green-Blue (RGB) color model sampled from the skin at the fin junction. We scored lateral barring following the same methodology used on preserved specimens (described above). Following tissue fixation in formalin and alizarin red staining following the protocol of Peichel et al. 2001, the fish were re-photographed and scored for melanophore density and lateral barring. A Pearson correlation test was used for both traits in order to assess if there was a meaningful relationship between pigmentation estimates obtained from stained versus living fish. A negative (melanophore density vs brightness) or positive correlation (lateral barring) between pre and post-staining measurements (at *p* < 0.05) indicated our stained pigment measurements indeed captured a meaningful aspect of biological reality.

### QTL mapping

The underlying genotype data was generated for and used in a previously described experiment (Rennison et al. 2019). DNA was extracted from each F_2_ individual’s fin clip (*N* = 400, 50 from each pond) using a standard phenol-chloroform extraction protocol. DNA was also extracted from the F_1_ parents and F_0_ grandparents. Libraries were prepared using the *PstI* enzyme following a genotyping by sequencing protocol (*as in* (Elshire et al. 2011)) and sequenced on an Illumina Hi-Seq 2000 platform. A standard reference-based bioinformatics pipeline was used to identify sequence variants (single nucleotide polymorphisms, SNPs) (see archived code from Rennison et al. 2019 for full details). Briefly, after demultiplexing, Trimmomatic (Bolder et al. 2014) was used to filter out low-quality sequences and adapter contamination. Reads were aligned to the stickleback reference genome (Jones et al. 2012) using BWA v0.7.9a (Li and Durbin 2009) with subsequent realignment using STAMPY v1.0.23 (Lunter and Goddson, 2011). For genotyping the GATK v3.3.0 (McKenna et al. 2010) best practices workflow (DePristo et al. 2011) was followed except that the MarkDuplicates step was omitted. RealignTargetCreator and IndelRealigner were used to realign reads around indels and HaplotypeCaller identified single nucleotide polymorphisms (SNPs) in individuals. Joint genotyping was done across all individuals using GenotypeGVCFs. The results were written to a single VCF file containing all variable sites. This file was filtered for a minimum quality score (of 20) and depth of coverage (minimum of 8 reads and maximum of 100,000) before use in downstream analyses.

A pedigree was built using the MasterBayes R package and a set of 1799 SNPs, which had minimal (<10%) missing data across all individuals. In order to have fully informative markers, only SNPs that were homozygous for alternative alleles in the benthic and limnetic grandparents of each F_2_ cross were used. These SNPs were then used to calculate pairwise recombination frequencies and create a genetic map using JoinMap version 3.0 (Ooijen and Voorrips, 2001). In total, 398 F_2_ progeny from the four F_1_ crosses were used for mapping, with many F_2_ families. F_2_ genotypes were coded according to the population code for outbred crosses, allowing segregation of up to four alleles per locus (cross-pollinator). The JMGRP module of JoinMap was used with a LOD score threshold of 4.0 to assign 2243 loci to 33 linkage groups. For each linkage group, a map was created with the JMMAP module. Mapping was done using the Kosambi function with a LOD threshold of 1.0, recombination threshold of 0.499, jump threshold of 5.0, and no fixed order. Two rounds of mapping were performed, with a ripple performed after each marker was added to the map.

QTL mapping was performed using Haley-Knott regressions across the F_1_ families (all-family QTL) and individual sex and family were set as covariates in the R/qtl package (Broman and Sen 2009) with 2037 SNP markers spread across the 21 chromosomes. The marker density was on average 4.2 SNP markers per kilobase (kb) (mean range across chromosomes 2.3 – 5.9 SNPs/kb). To test if there was any variation among the four F_1_ families in the genetic basis of divergent pigmentation, we also performed QTL mapping independently for each family (within-family QTLs) with sex as a covariate, and sample sizes of 93–99 individuals per F_1_ family. We calculated the percentage variance explained (PVE) for each candidate locus using the equation: PVE = 1 – (10^(−2*(LOD/n))), where LOD is the estimated LOD score and n is the sample size. The significance threshold for each phenotype was estimated using permutation testing with 10,000 iterations. Overall phenotypic heritability for the two pigmentation traits was calculated by estimating a kinship matrix using the *kinship2* R package and the *est_herit* function in the *qtl2* package.

### Functional enrichment testing

We identified candidate genes from within the 1.5 LOD interval region surrounding the QTL peaks; the resulting candidates were then explored through comparisons to existing candidates from other taxa and using functional enrichment testing. First, we determined whether any of the candidate loci found within our QTL peaks had been previously associated with pigmentation in other vertebrates. To this end, we determined what genes fell within the chromosomal regions associated with the significant peaks in the all-family QTL. We searched for matches between our candidate gene list and pigmentation loci using previously curated and published pigmentation lists (Baxter et al. 2019; McKinnon et al. 2022). Second, we performed functional enrichment of Gene ontology terms associated with the candidate gene list to assess if any functional pathways were overly represented. We used the biomaRt package to extract gene position, gene IDs, HGNC symbols, and associated gene ontology (GO) terms from the stickleback genome (Durinck et al. 2009). The GO pathways associated with each gene ID of interest in our study were extracted and topGO was used to test for functional enrichment across our set of candidates (Alexa and Rahnenführer 2023). To test for significant enrichment, a Fisher’s exact test based on annotated gene counts was run that took into consideration GO hierarchy, and the algorithm was set to weight01. Significant GO terms were visualized using ‘showSigOfNodes’ and we identified which and how many genes in our candidate gene list were associated with these pathways.

## Results

### Pigmentation variation

**Fig. 2:**
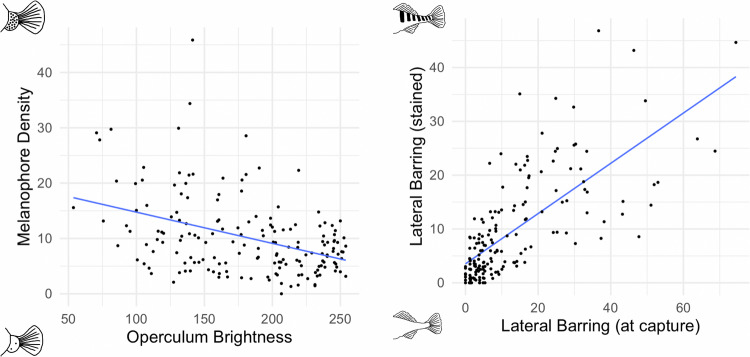
Pigmentation phenotypes in stained stickleback as they relate to pigmentation in non-stained living fish.

The pigmentation phenotypes characterized in our F_2_ mapping population of stained fish appeared to capture a biologically relevant pigment trait present in live wild-caught fish (Fig. 2). There was evidence of a significant negative correlation between melanophore density of stained fish and brightness at the fin junction measured from living fish (*r* = −0.41, *p* < 0.0001). Thus, the “brighter” the operculum (i.e., the less dark), the lower the melanophore density. There was also a significantly positive correlation between lateral barring in living and stained fish (*r* = 0.71, *p* < 0.0001). The strength of the relationship between these variables varied amongst the populations for both lateral barring (0.63 to 0.36) and melanophore density (−0.26 to −0.54).

**Fig. 3:**
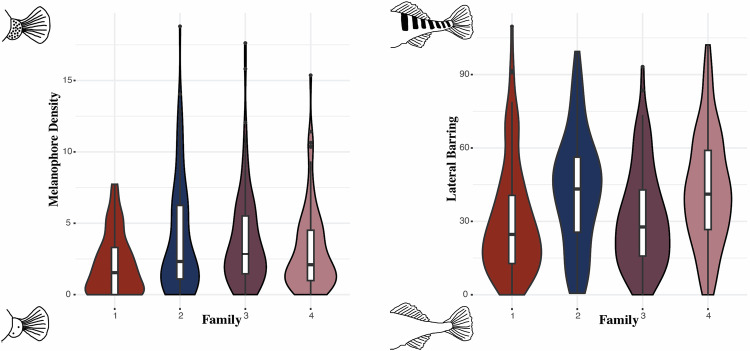
Distribution of melanophore density (number of visible melanocytes on the fin junction) and lateral barring (difference in light and dark patches on the ventral flank) across F_1_ families of benthic-limnetic hybrids used for QTL mapping.

Within the benthic-limnetic F_2_ hybrid individuals used for QTL mapping, we did not find any relationship between the two surveyed pigmentation traits (melanophore density and lateral barring) (*r* = 0.01; p > 0.05). This suggests that these phenotypes are largely independent of each other. Across the F_1_ mapping families there were statistically significant differences in melanophore density (F = 6.79; *p* < 0.0001) and lateral barring (F = 9.45; *p* < 0.0001). In general, families two and three exhibited greater melanophore density at the fin junction, while families two and four displayed more lateral barring (Fig. 3).

### QTL mapping

#### Melanophore density

**Fig. 4:**
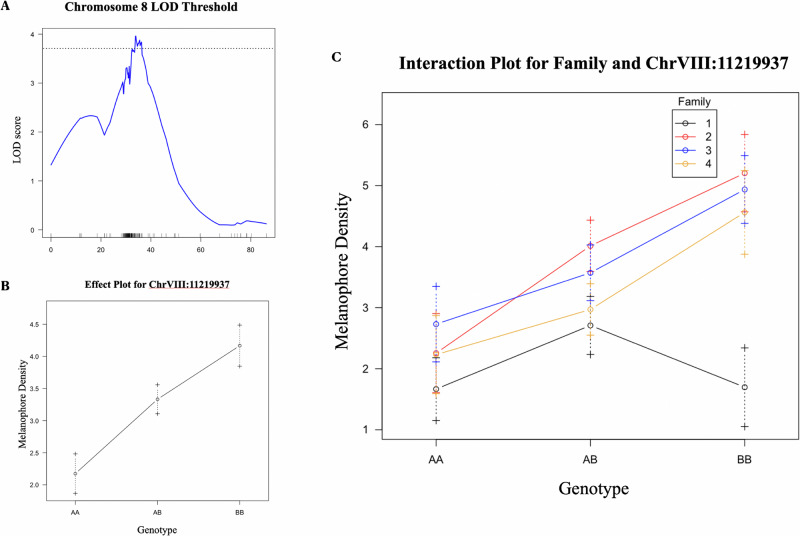
QTL mapping of melanophore density in F_2_ benthic-limentic hybrid fish. **A** LOD plot for chromosome eight candidate for melanophore density in the all-family analysis, **B** effect plot for chromosome eight candidate peak, and **C** interaction plot across the four F_1_ families.

When mapping melanophore density across all four F_1_ families, there was a single significant QTL peak, which was located on chromosome 8 (Figs. 4A, S6). Examination of the 1.5-LOD support interval placed the peak in a region encompassing ≈8 Mb, between markers ChVIII:7120492 and ChVIII:14925951. The alleles at this QTL had predominantly additive effects (Fig. 4B), although when family four was analyzed independently it exhibited a non-additive effect (Fig. 4C). Across families, this QTL explained ~5% of the total phenotypic variance. Within each family the variance explained ranged from 7.4 – 10.2% (Family 1 = 7.42%; Family 2 = 10.21%; Family 3 = 7.85%; Family 4 = 9.03%). QTL mapping conducted *within* each F_1_ family identified an additional peak in family 1 for melanophore density on chromosome 18 between markers ChXVIII:2652629 and ChXVIII:12506627 (Table 1) that explained 9% of the phenotypic variance. The total heritability of this trait was 0.28 when estimated across all families and markers.

**Table 1:** Highest significant candidate peaks associated with melanophore density.

Sample	Chr	LOD	cM	Marker near peak	PVE
*All Family*	8.2	3.96^a^	33.99	chrVIII:11219937	5.18
*Family 1*	18	3.79^a^	44.99	chrXVIII:8361341	9.61

^a^indicates the peak passes the 5% significance threshold as determined by permutation.

#### Lateral Barring

**Fig. 5:**
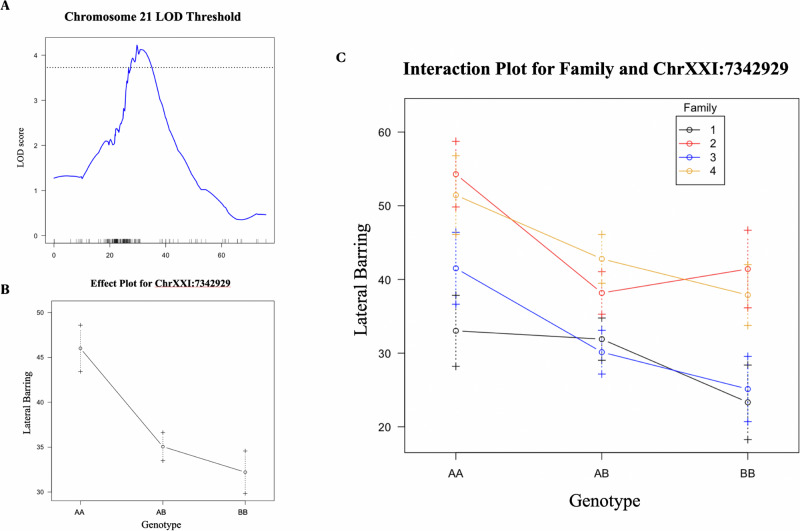
QTL mapping of lateral barring in F_2_ benthic-limnetic hybrid fish. **A** LOD plot for chromosome 21 for lateral barring in the all-family analysis, **B** effect plot for chromosome 21 candidate peak, and **C** interaction plot across the four families.

In the all F_1_ family QTL analysis for lateral barring, a significant candidate peak was identified on chromosome 21. Examination of the 1.5-LOD support interval places the peak in a region encompassing ≈7 Mb, between markers chrXXI:3061663 and chrXXI:10192631 (Figs. 5A, S7). Similar to mapping for melanophore density, the effects were additive in the all-family analysis (Fig. 5C). The overall percent variance explained was 4.26%, and phenotypic variance explained again differed among families ranging from 4.5 – 11% (Family 1 = 4.62%; Family 2 = 11.03%; Family 3 = 11.24%; Family 4 = 5.22%). One additional candidate on chromosome 16 was identified in the F_1_ family 2 analysis (see Table 2). The total heritability of this trait was 0.35 across all families and markers.

**Table 2:** Highest significant candiate peaks associated with lateral barring.

Sample	Chr	LOD	cM	Marker near peak	PVE
*All Family*	21^a^	4.22	29.7	chrXXI:7342929	4.26
*Family 2*	16^a^	4.70	63.12	chrXVI:563523	19.45

^a^indicates the peak passes the 5% significance threshold determined through permutation.

### Functional enrichment

Across both traits, we identified 1,099 loci associated within significant QTL intervals in both the all-family and within-family QTLs. Across the all-family crosses, there were 638 genes within the 1.5 LOD interval across both outlier chromosomal regions, and approximately 5% were previously identified pigmentation genes. Among the candidates resulting from our all-F_1_ family map of melanophore density, we identified 350 candidate genes, and 11 of these genes (~4%) were previously shown to be associated with pigmentation in other vertebrates. For lateral barring, we identified 288 candidates in the all-family cross, and of these, 8 genes (~3%) were previously known vertebrate pigmentation loci.

To further investigate our set of candidates, we performed functional enrichment testing separately on candidate regions on chromosomes eight and twenty-one, for melanophore density and lateral barring, respectively. Using the lists of 140 and 119 annotated candidate genes we identified 22 significantly enriched pathways (Table 3). From the annotated chromosome 8 candidates, there were 8 enriched pathways: five involved in catalytic activity, one in transporter activity, one in binding, and the last in molecular function regulation (S8). Among the chromosome 21 candidates, there were 14 pathways significantly enriched: three involved in binding, and the rest involved with catalytic activity (S9). Within these enriched pathways, three of the genes have been previously associated with pigmentation pathways (Table 3).

**Table 3:** Significantly enriched pathways and genes associated with each chromosome.

Pathway name	GO	Sig.	Expected	Fis	Genes
*Chromosome 21- melanophore density*					
Carboxypeptidase activity	0004788	2	0.16	0.011	*cpa6, cpb1*
Protein kinase activity	0003834	9	8.11	0.013	*mak, sgk3, cdk8, acad11, yes1, fastkd3, map3k22*
Beta-carotene 15,15’-dioxygenase activity	0004864	1	0.01	0.015	***bco1***
Thiamine diphosphokinase activity	0004333	1	0.01	0.015	*tpk1*
Thiamine triphosphate phosphatase activity	0050333	1	0.01	0.015	*thtpa*
Glycine-tRNA ligase activity	0004820	1	0.01	0.015	*gars1*
Nuclear thyroid hormone receptor binding	0001882	1	0.01	0.015	*ncoa2*
Thymidylate synthase activity	0046966	1	0.01	0.015	***tyms***
Protein dimerization activity	0004180	5	1.45	0.033	*msc, ncoa2, TCF24, bhlhe22, myca*
Deoxyribodipyrimidine photo-lyase activity	0004652	1	0.03	0.029	*cry-dash*
Methylated-DNA-[protein]-cysteine S-meth	0003779	1	0.04	0.044	*klhl40b*
Sulfuric ester hydrolase activity	0008484	1	0.04	0.044	***sulf1***
Hydroxymethylglutaryl-CoA reductase	0004420	1	0.04	0.044	*gdap1*
*Chromosome 8 – lateral barring*					
Furmarate hydratase activity	0004333	1	0.02	0.017	*fh*
Protein phosphatase inhibitor activity	0004864	1	0.02	0.017	*pp1r2*
Protein tyrosine kinase activity	0004713	17	9.27	0.021	*tie1, ror1, jak1, zap70, mknk2b, csnk1g2b, nek7, mast3b, jak3, EPHB3, obscna, matk, abl2*,
Galactosyltransferase activity	0008378	2	0.30	0.035	*b3gnt3.4, b3gnt7*
Thiol oxidase activity	0016972	1	0.03	0.035	*lhx4*
Coproporphyrinogen oxidase activity	0004109	1	0.03	0.035	*cpox*
Lipid transporter activity	0005319	1	0.05	0.051	*vtg3*
Histone binding	0042393	1	0.05	0.051	*uhrf1*

Key: Genes with known pigmentation associations indicated in bold.

## Discussion and Conclusion

We investigated the genetic basis of two melanin pigmentation phenotypes, lateral barring and melanophore density, using a large sample of benthic-limnetic F_2_ hybrid stickleback. QTL mapping was conducted across and within families; the resulting candidates were compared to known pigmentation genes and their functions were explored using GO term analysis. Using this approach, we found that the two pigmentation traits were uncorrelated and mapped to distinct genetic regions (Figs. 4A and 5A), suggesting they are independent traits. Melanocyte density mapped to chromosome 8 (Fig. 4A), and degree of lateral barring mapped to chromosome 21 (Fig. 5A). A previous QTL study of marine-freshwater stickleback crosses found candidates for melanization of the gills and ventral flank map to the *kitlg* locus on chromosome 19 (Miller et al. 2007). Prior work examining the degree of lateral barring in marine-freshwater crosses identified candidate regions on chromosomes 1, 6, and 11 (Greenwood et al. 2011). This suggests that distinct genes may underly different components of melanism across the body and/or that the genetic architecture of lateral barring may differ between marine-freshwater populations relative to benthic-limnetic populations. Alternatively, the distinct loci underlying lateral barring in marine-freshwater pairs could be due to a failure to detect small effects in the benthic-limnetic crosses. Our results indicate that the effects on the QTL were additive (Figs. 4B and 5B), which is in line with previous findings (Miller et al. 2014). The identified loci are of relatively small effect, suggesting that these traits are likely polygenic.

Large effect loci have been identified for several key ecological traits in stickleback including lateral plate count (>76% variance explained), neuromast pattern (>39% variance explained) and pelvic spine length ( > 65% variance explained) (Colosimo et al. 2005; Erickson et al. 2016; Wark et al. 2012). Yet, small effect loci also contribute to these traits, and variance in other important stickleback traits, including other defense traits (e.g. dorsal spine length), trophic traits (e.g. gill raker number and length, tooth number), and body shape have been shown to have a highly polygenic architecture with many loci of relatively small effect contributing to phenotypic variation (Erickson et al. 2016; Miller et al. 2014; Peichel and Marques 2017). Prior work on melanization and degree of barring found a combination of relatively small effect loci (6.6–11.7% variance explained) and moderate effect loci (~20% variance explained) (Greenwood et al. 2011; Greenwood et al. 2012). In contrast, a large effect locus, *kitlg*, explains >56% of the variance in gill melanic pigments in marine-freshwater (Miller et al. 2007). Variability in the complexity of the architecture of pigmentation has also been found in other taxa. For example, in *Peromyscus* mice and several lizard species, differences in pigmentary loss or gain has been attributed to a single mutation (Hoekstra et al. 2006; Nachman et al. 2003; Rosenblum et al. 2010), while in *Drosophila*, the degree of melanization is often associated with a suite of small effect genes (Dembeck et al. 2015).

Within a single species, differences in genetic background can impact the phenotype through epistatic interactions. For example, in beach mice, lighter coloration associated with one gene (*mc1r*) is not apparent unless another gene (*asip*) also increases its expression (Steiner et al. 2008). The phenotypic and genetic effects of pigmentation loci will thus vary among and between populations and species (Hubbard et al. 2010; Manceau et al. 2010). Our results indicate the variation between families in their expressed phenotypes, effect sizes, and dominance effects (Figs. 3, 5C), which could be due to epistasis. A similar pattern of family-level variation was detected previously when mapping skeletal traits in benthic-limnetic F_2_ crosses (Rennison et al. 2019). The observed variation in mapped QTLs among F_1_ families could result from the presence of different segregating variances present in the pure benthic and limnetic parents used for each F_1_ cross. Alternatively, the variation in F_1_ families may be a result of stochastic differences in the power of detection of these relatively small effect loci. With only 100 individuals per family, loci near the significance cut-off could fall just above the threshold in one family and below in another. Differences in the fraction of missing data across individuals for each family could also contribute to the pattern of variable detection. Unfortunately, due to the absence of inter F_1_ family crosses and the relatively small sample sizes of the individual families we did not have the power or experimental framework to investigate these potential epistatic effects in this experiment.

From the candidate QTL regions for these two pigmentation traits, several new candidate pigmentation genes were identified for benthic and limnetic stickleback (S6). Three of the candidate loci (*sulf1*, *bco1*, *tyms*) were associated with functionally enriched pathways (Table 3). Of these, *bco1* has been previously associated with fish carotenoid pigmentation, including in threespine stickleback (Huang et al. 2021; McKinnon et al. 2022). Another candidate, *tyms*, is known to lead to abnormal pigmentary patterns in zebrafish (Amsterdam et al. 2004; *Phenotype Annotation (1994–2006)*, 2006). While all three of these genes are associated with pigmentation phenotypes in other vertebrates, only one is associated with other stickleback pigmentation phenotypes. Of the genes found proximate to our QTL peaks, >500 had no prior known role in pigmentation and only 30 (~5.5%) were functionally enriched. Thus, our survey expands this candidate list of potential loci underlying pigmentation evolution.

This study demonstrates that quantification/characterization of melanic pigmentation in stained stickleback provides a likely functionally relevant estimation of melanic traits in living fish. Previous pigmentation work on stickleback has been largely limited to phenotyping conducted using photos from living fish, specimens phenotyped immediately following euthanasia, or surveys of internal structures (Greenwood et al. 2011; Malek et al. 2012; McKinnon et al. 2022; Yong et al. 2015). Our finding that pigmentation phenotypes collected from preserved and stained specimens produce biologically meaningful data opens up additional opportunities to study pigmentation using museum collections or in instances where live photographs would be difficult to collect. Stickleback pigmentation genetics remains understudied relative to other traits (Reid et al. 2021), and fish pigmentation genetics is generally understudied relative to other vertebrates (Elkin et al. 2022). This is likely due in part to pigmentation being more challenging to quantify in fish than in other vertebrates. For example, while mammals have only one type of chromatophore, fish have six different kinds (black melanophores, yellow-orange xanthophores, red erythrophores, light-reflecting iridophores, white leucophores, blue cyanophores) (Cal et al. 2017; Kelsh 2004). Many chromatophores exhibit plasticity to environmental conditions (e.g., red erythrophores influenced by carotenoid availability in the diet (Pike et al. 2011). However, the extent to which pigment phenotypes can change depends on the concentrations of melanosomes (Logan et al. 2006). As such, melanic traits may be more static and thus more easily phenotyped. As the list of pigmentation QTLs grows for threespine stickleback—a model in evolutionary biology—it can aid us in understanding the predictability of phenotypic and genotypic evolution and the origins of adaptive genetic variation.

### Data archiving

Underlying data and code are archived on GitHub: https://github.com/djrennison/Heredity_pigment.

## Supplementary information

The genetic basis of divergent melanic pigmentation in benthic and limnetic threespine stickleback
