# Supplementary material for: The genetic basis of divergent melanic pigmentation in benthic and limnetic threespine stickleback
Source: Heredity (Edinb). 2024 Jul 24;133(4):207–15. doi: 10.1038/s41437-024-00706-0 (PMC11437277; doi:10.1038/s41437-024-00706-0)
Supplement: Supplementary file 1 — The genetic basis of divergent melanic pigmentation in benthic and limnetic threespine stickleback [file 41437_2024_706_MOESM1_ESM.pdf]

**S1.** Aerial photograph of the experimental pond facility, indicating length and width. Humans are pictures adjacent to the pond for scale. Photo courtesy of Thor Veen.

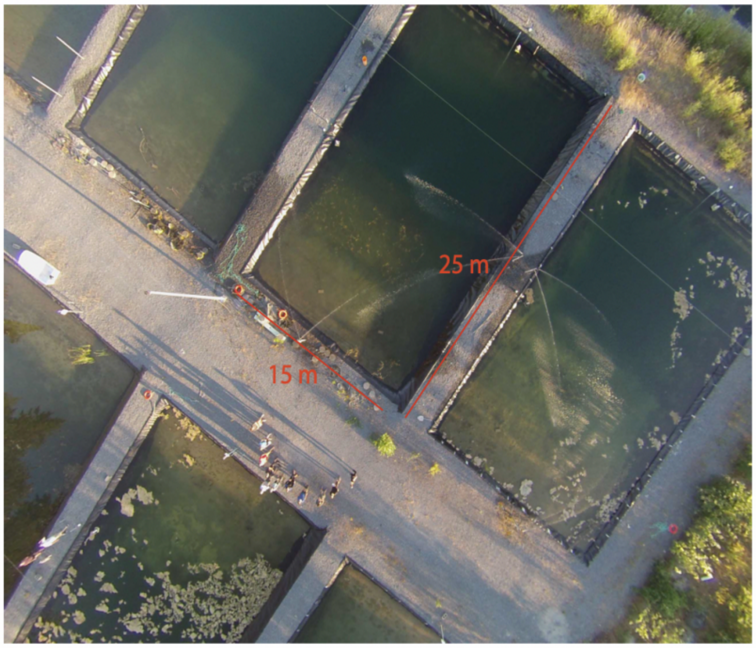


**S2.** Experimental setup and timeline. A shared color between ponds indicate they had the same founders.

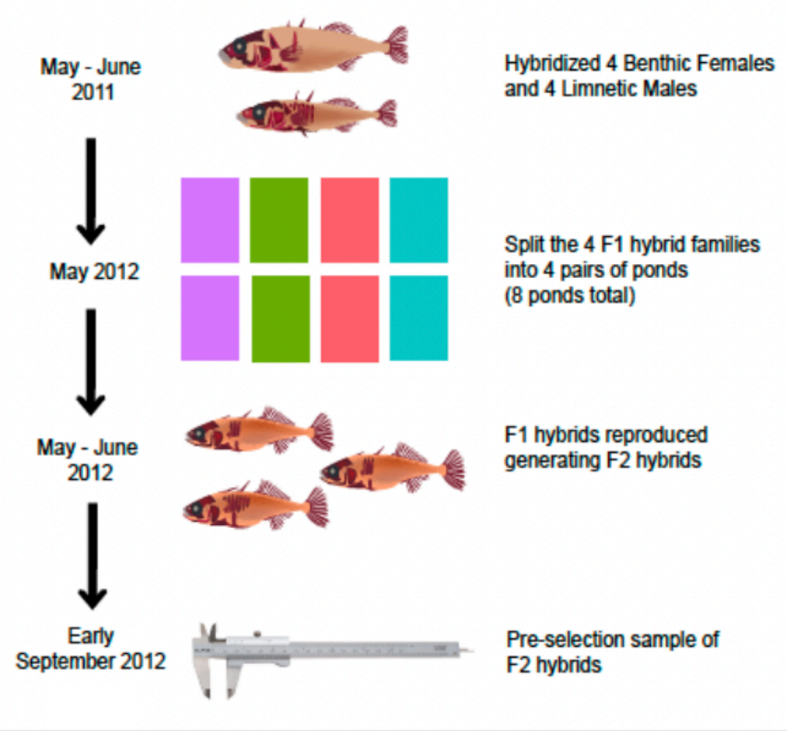

**S3.** Melanocyte counting at the fin junction

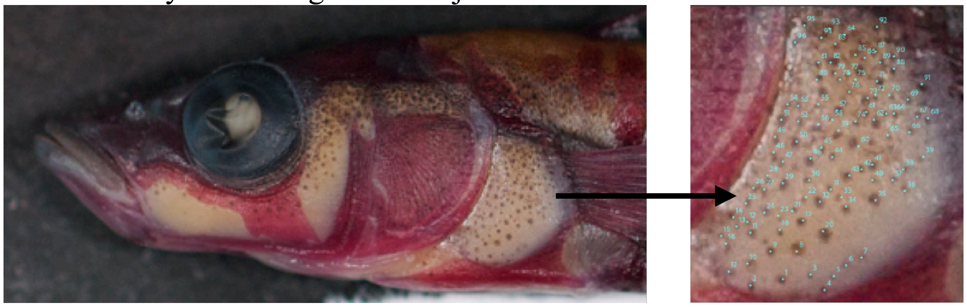

**S4.** Lateral barring at the ventral flank

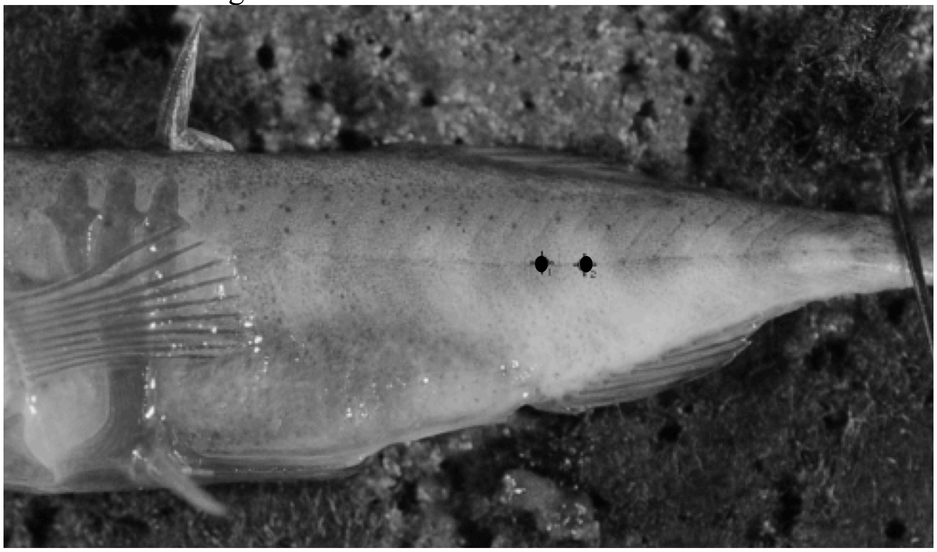

**S5.** Marine stickleback photographed against an X-rite color checker passport

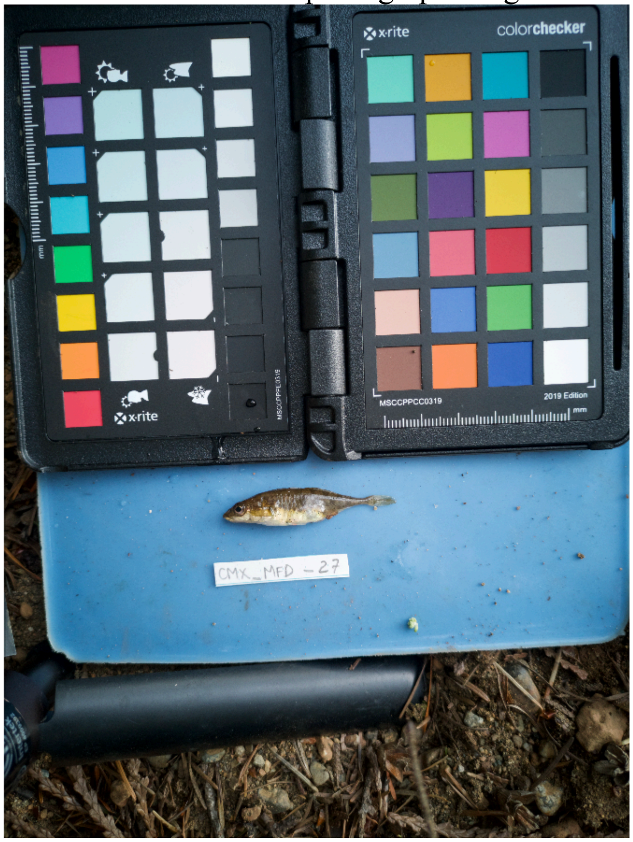


**S6** LOD plot for all chromosomes in the all-family analysis covariate-controlled analysis of melanophore density. Horizontal bar indicates LOD significance threshold as estimated from 10,000 permutation iterations.

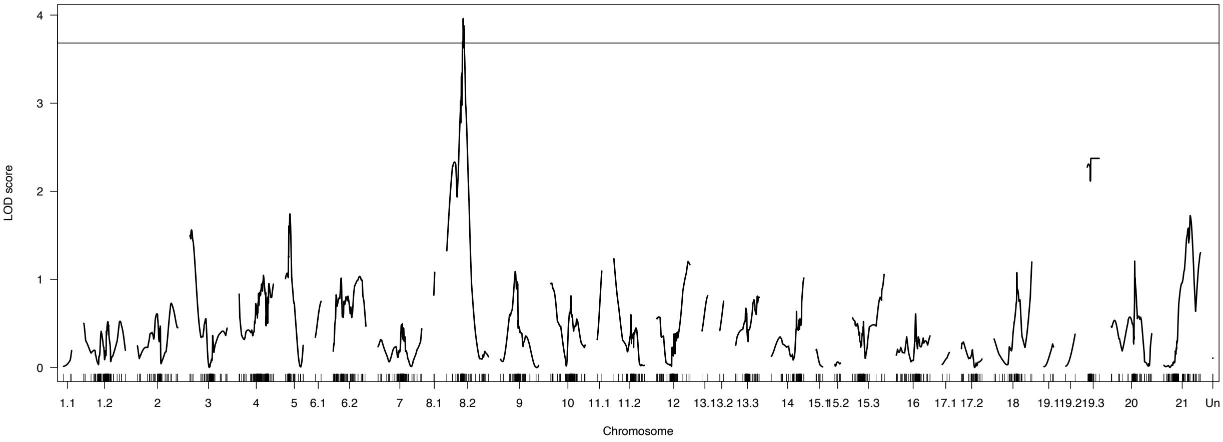

**S7** LOD plot for all chromosomes in the all-family analysis covariate-controlled analysis of lateral barring. Horizontal bar indicates LOD significance threshold as estimated from 10,000 permutation iterations.

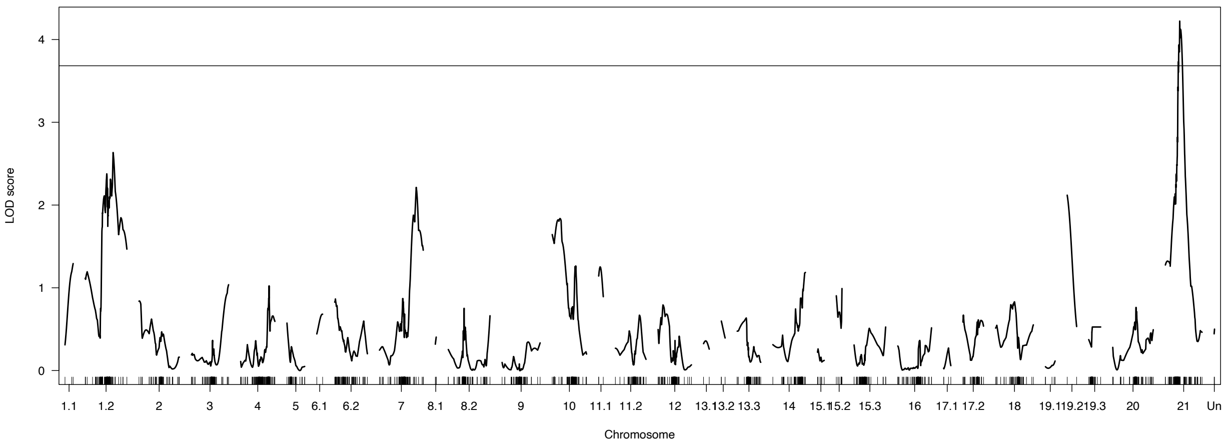

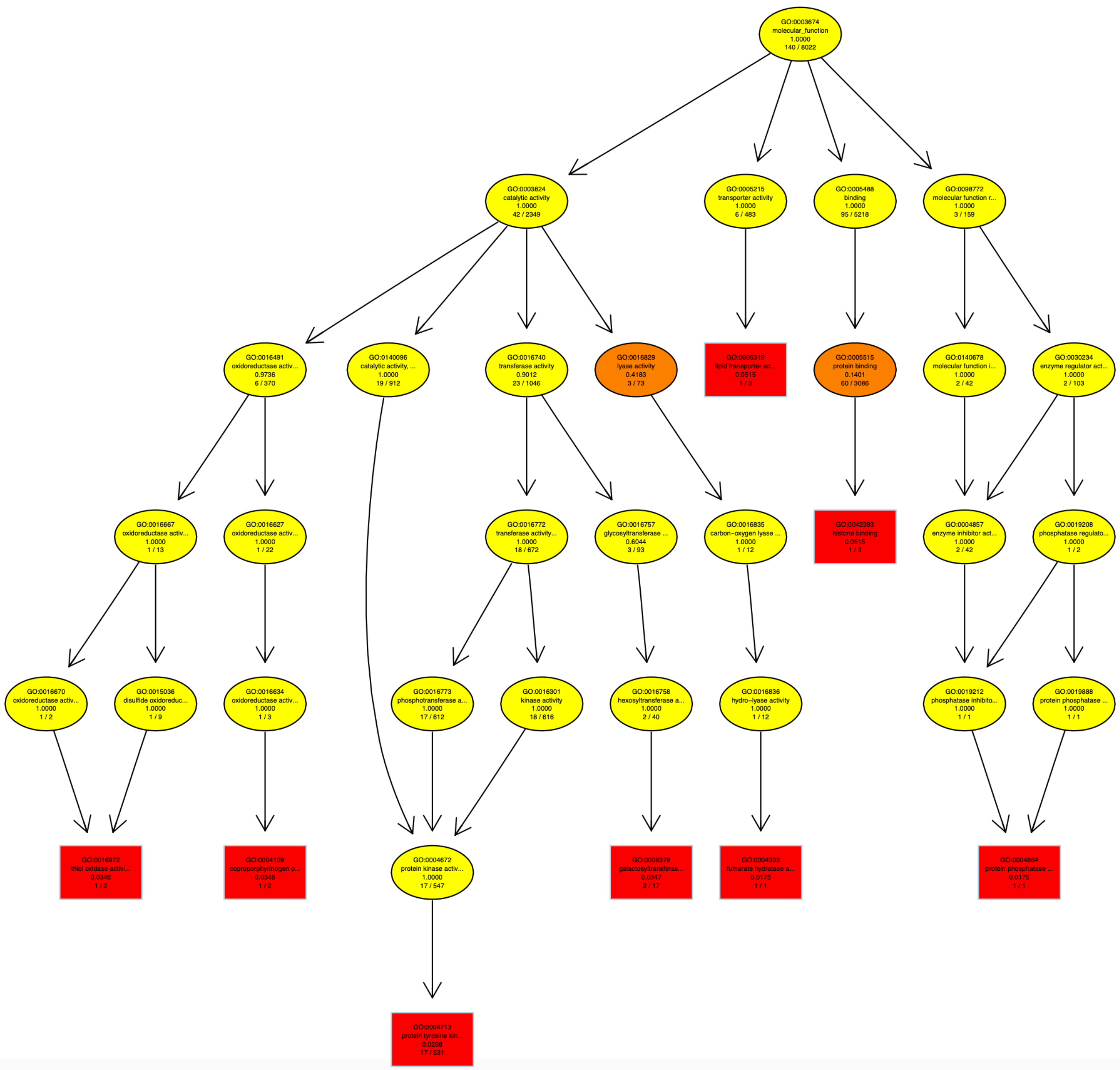


**S8** Significantly enriched metabolic pathways associated with melanophore density. Gene ontology terms highlighted in red had a P-value < 0.01.

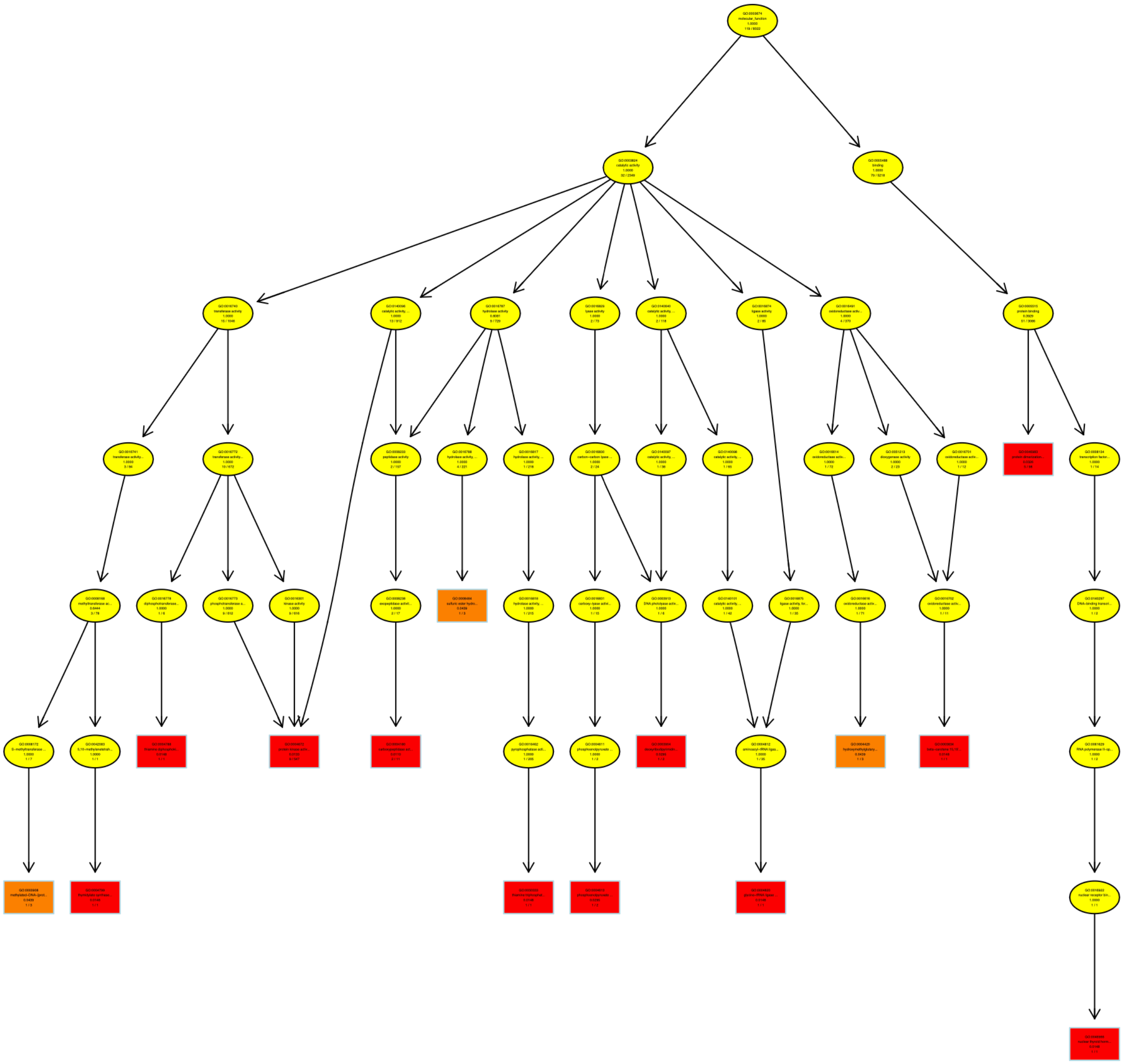

**S9** Significantly enriched metabolic pathways associated with lateral barring. Gene ontology terms highlighted in red had a P-value < 0.01.
